# Predicting drug targets by homology modelling of *Pseudomonas aeruginosa* proteins of unknown function

**DOI:** 10.1371/journal.pone.0258385

**Published:** 2021-10-14

**Authors:** Nikolina Babic, Filip Kovacic

**Affiliations:** Institute of Molecular Enzyme Technology, Heinrich-Heine Universität Düsseldorf, Forschungszentrum Jülich GmbH, Jülich, Germany; Laurentian University, CANADA

## Abstract

The efficacy of antibiotics to treat bacterial infections declines rapidly due to antibiotic resistance. This problem has stimulated the development of novel antibiotics, but most attempts have failed. Consequently, the idea of mining uncharacterized genes of pathogens to identify potential targets for entirely new classes of antibiotics was proposed. Without knowing the biochemical function of a protein, it is difficult to validate its potential for drug targeting; therefore, the functional characterization of bacterial proteins of unknown function must be accelerated. Here, we present a paradigm for comprehensively predicting the biochemical functions of a large set of proteins encoded by hypothetical genes in human pathogens to identify candidate drug targets. A high-throughput approach based on homology modelling with ten templates per target protein was applied to the set of 2103 *P*. *aeruginosa* proteins encoded by hypothetical genes. The >21000 homology modelling results obtained and available biological and biochemical information about several thousand templates were scrutinized to predict the function of reliably modelled proteins of unknown function. This approach resulted in assigning one or often multiple putative functions to hundreds of enzymes, ligand-binding proteins and transporters. New biochemical functions were predicted for 41 proteins whose essential or virulence-related roles in *P*. *aeruginosa* were already experimentally demonstrated. Eleven of them were shortlisted as promising drug targets that participate in essential pathways (maintaining genome and cell wall integrity), virulence-related processes (adhesion, cell motility, host recognition) or antibiotic resistance, which are general drug targets. These proteins are conserved in other WHO priority pathogens but not in humans; therefore, they represent high-potential targets for preclinical studies. These and many more biochemical functions assigned to uncharacterized proteins of *P*. *aeruginosa*, made available as PaPUF database, may guide the design of experimental screening of inhibitors, which is a crucial step towards the validation of the highest-potential targets for the development of novel drugs against *P*. *aeruginosa* and other high-priority pathogens.

## Introduction

Even after more than a half-century of development of antibiotics, bacterial infections remain a significant cause of morbidity and mortality worldwide for various reasons including unsuccessful antibiotic treatments. The rise in multidrug-resistant bacterial infections has been further accelerated by the overuse of antibiotics in present pandemic conditions [1]. It is evident that without new countermeasures against multidrug-resistant bacteria, so-called “superbugs”, routine medical procedures (cancer chemotherapy, organ transplantation, diabetes treatment) become very high risk, and the achievement of the Millennium Development Goals is endangered [2].

Attempts of the World Health Organization (WHO) to guide and promote research and development (R&D) to obtain new antibiotics failed [2, 3]. The hurdles faced by the current system for the R&D of antibiotics are tremendous, which is the reason why only a few solutions (32 antibiotics and ten biological treatments) the WHO priority pathogens are currently in the R&D pipeline (clinical phases 1 to 3) [2–4]. Generally, the most attractive targets for new classes of antibiotics are proteins involved in previously untargeted essential biochemical pathways that are conserved among pathogens and absent in humans [1, 5]. The targeted screening of such enzymes identified the reductase IspH, which is involved in isoprenoid biosynthesis [6]; the reductase FabI, which is involved in fatty acid biosynthesis [7]; and the peptidase LspA, which is involved in the posttranslational processing of lipoprotein [8]. The inhibition of these enzymes by C23.28–TPP (preclinical study) [6], isoniazid (approved drug) [7] and myxovirescin (clinical phase 3) [8], respectively, showed strong antibacterial activity.

New antibiotics represent only one solution with uncertain efficacy because of the fast evolution of resistance observed after each new antibiotic was introduced [9]. Another promising strategy is to counteract the nonessential functions mediated by bacterial virulence or antibiotic resistance factors, thereby blocking host-pathogen interactions, the progression of infection, or antibiotic expulsion without killing the bacteria or inhibiting their growth. Such antivirulence drugs, suggested as a promising supplement with the ability to enhance the effects of classical antibiotics, are thought to exert weak selective pressure, minimizing the risk of resistance emergence [10]. The main antivirulence approaches of drugs in clinical development include the inhibition of quorum-sensing systems, biofilm formation, and toxin production or function [7, 10].

Obviously, several proteins targeted by novel potential antibiotics or antivirulence drugs were identified in the last decade; however, many more novel essential proteins or important virulence factors with unknown functions in bacteria still need to be characterized, as it is difficult to set up an inhibitor screening for proteins of unknown function (PUFs) [1]. Therefore, understanding the function of these proteins is a prerequisite for the validation of targets for the development of antibacterial drugs with novel mechanisms of action.

*P*. *aeruginosa*, a critical priority pathogen according to the WHO, is a serious threat in hospitals, where multidrug-resistant strains cause severe acute infections, often with deadly outcomes [3]. Life-threatening chronic *P*. *aeruginosa* infections have been frequently observed in immunocompromised persons, e.g., cystic fibrosis and cancer patients [11]. *P*. *aeruginosa* infections are a concern because of the high resistance towards the best currently available antibiotics—carbapenems and the third generation of cephalosporins [3]. To cope with antibiotics, *P*. *aeruginosa* uses various intrinsic and acquired resistance mechanisms, including the expression of different classes of β-lactamases, the loss of outer membrane channel proteins to increase outer membrane impermeability, the overexpression of antibiotic efflux pumps, the synthesis of antibiotic modifying enzymes, and mutations in the primary targets of quinolone antibiotics [12]. Moreover, the formation of antibiotic-resistant biofilms [12], exceptional adaptability to infect various human tissues owing to multiple signalling pathways [13] and cell surface adhesins [14, 15], and the fast progress of infections as a result of the production of a vast number of potent extracellular toxins [16] present challenges in the treatment of *P*. *aeruginosa* infections. All these features qualify *P*. *aeruginosa* as a model pathogen bacterium for the R&D of novel antibiotics.

Here, we aimed to assign biochemical functions to hypothetical proteins from the human pathogen *P*. *aeruginosa*. Using a homology modelling-based high-throughput approach, novel putative functions were reliably assigned to many proteins for which functions could not be previously predicted from the sequence. Several proteins with new putative biochemical functions could be linked to important infection-related processes, which suggests that these proteins are promising targets for the development of drugs against *P*. *aeruginosa*.

## Materials and methods

### Selection of target genes

The GUFs from *P*. *aeruginosa* PAO1 were defined as genes with “hypothetical protein” and “conserved hypothetical protein” annotations in the product name field of the *Pseudomonas* Genome Database (PGD) [17]. In the PGD, all genes were analysed by sequence alignment and classified into four classes. Class IV includes hypothetical proteins, which are defined as those proteins whose function could not be proposed based on conserved amino acid motifs, structural features, or limited sequence similarity with experimentally studied proteins. Gene and protein sequences were retrieved from the PGD (www.pseudomonas.com) and UniProt databases [18] (www.uniprot.org), respectively.

### Comparative genomics

PGD annotations were used to find proteins with human homologues, proteins with homologues in other pathogens, and virulence factors. The list of virulence factors was obtained from the Virulence Factor Database (www.mgc.ac.cn/VFs) [19] and the Victors Virulence Factors Knowledge Database (www.phidias.us/victors) [20]. Comparative BLAST was performed using NCBI’s BLAST suite (https://blast.ncbi.nlm.nih.gov/Blast.cgi).

A Diamond BLASTp tool [21] from the PGD was used to search for groups of orthologues with >70% sequence identity and >95% alignment coverage among different *P*. *aeruginosa* strains isolated from humans with developed disease as indicated in the PGD and among other pathogenic *Pseudomonas* species, namely, *P*. *alcaligenes*, *P*. *mendocina*, and *P*. *otitidis*. NCBI BLAST was used to search for homologues with >35% sequence identity and >75% alignment coverage in 13 WHO priority pathogens.

### Homology modelling

Homology modelling was performed using the batch processing feature of the Phyre2 web server [22, 23]. The obtained output file was imported to Microsoft Excel, and the information on the ten best structural models was analysed to identify reliable homology models. For this purpose, the following cut-off values were used: modelling confidence >75%, domain size >100 residues for transporters and enzymes, and domain size >50 residues for binding proteins. The functions of reliable templates were identified by text mining the information from the “PDB header” and “PDB molecule” fields in the Phyre2 output file. Text mining was performed automatically using Excel, and the keywords are listed in S6 Table.

Selected models were energy minimized several times with the GROMOS96 43B1 force field as implemented in Swiss-PdbViewer [24]. The stereochemical parameters of the homology models were evaluated using PROCHECK v.3.5 [25]. The DALI database [26] (http://ekhidna2.biocenter.helsinki.fi/dali/oldstyle.html) was used to identify structures similar to the homology models. The protein structures were analysed, aligned, and visualized using PyMOL (http://www.pymol.org).

## Results

### *P*. *aeruginosa* genes of unknown function are potential antibiotic targets

The development of efficient drugs against multiresistant bacteria relies on the identification of entirely new bacterial targets whose inhibition will hamper bacterial growth (antibiotics) or prevent the pathogen from being virulent (antivirulence drugs). Such targets are likely to be found among genes of unknown function (GUF), so-called genomic “dark matter” [1]. Interestingly, even 20 years after the *P*. *aeruginosa* PA01 genome was sequenced, nearly 40% of genes remain GUFs, which is true of the majority of sequenced bacterial genomes [27, 28].

**Fig 1:**
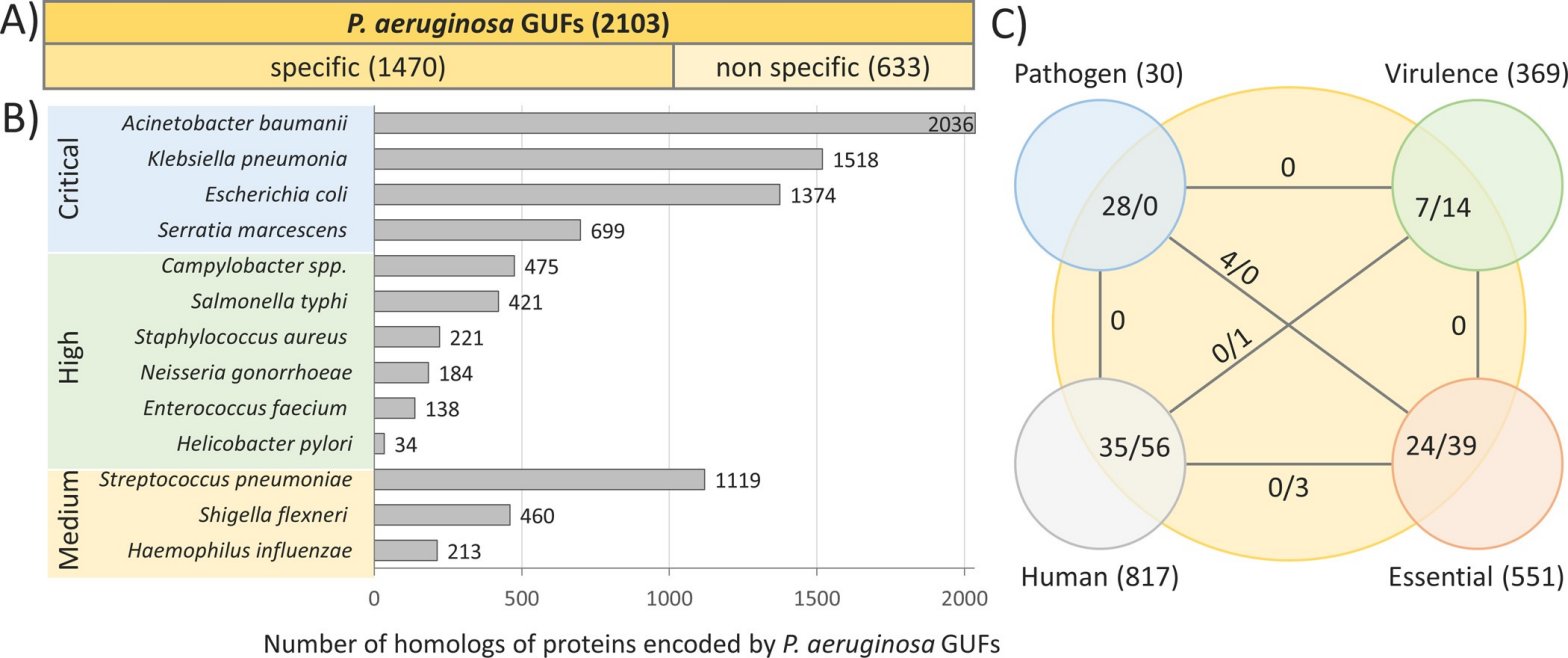
A large portion of the genomes of *P*. *aeruginosa* PA01 and WHO priority pathogens consist of GUFs. **A)** The majority of *P*. *aeruginosa* GUFs are found only in *Pseudomonas* species (specific), while some of them are conserved in other organisms (nonspecific). The analysis was performed using the PGD version **20.2** download on 5.11.2020. **B)** Conservation of *P*. *aeruginosa* genes of unknown function in WHO priority pathogens. *P*. *aeruginosa* PUFs were used as a query for a BLAST search on NCBI to find protein homologues (>35% sequence identity, >75% sequence coverage) in the following thirteen WHO priority pathogens. Taxonomic identifiers of organisms are listed in S2 Table. **C)** Pathogen-specific genes, essential genes, virulence factor genes and genes with human homologues among GUFs. Numbers in brackets indicate the number of genes in each group. A number of *P*. *aeruginosa* GUFs in overlapping sections is indicated as the number of specific genes/number of nonspecific genes. Numbers on lines indicate mutual GUFs among two linked groups.

To identify GUFs in *P*. *aeruginosa* PAO1, we searched for functional class IV hypothetical genes in the manually curated *Pseudomonas* Genome Database (PGD) [17]. The results revealed 1470 hypothetical genes, which have no homologues in other species, and 633 conserved hypothetical genes, which have homologues without known function in other bacteria (Fig 1A and S1 Table). These 2103 genes of *P*. *aeruginosa* considered GUFs were analysed by integrating comparative sequence analysis and structural homology-based functional prediction.

Bacterial antibiotic targets that are absent in humans are particularly interesting, as their inhibition should not exert side effects on host physiology [1, 7]. PGD [17] annotation of *P*. *aeruginosa* proteins with human homologues revealed that even 96% of GUFs do not have human homologues (Fig 1C).

The essential genes, genes conserved only in pathogenic species and genes with demonstrated virulence function represent high-priority target groups for the R&D of antibiotics because they may participate in key cellular or virulence processes whose inhibition might be lethal or virulence attenuating [29]. We found that 63 GUFs of *P*. *aeruginosa* are essential genes (Fig 1C and S1 Table), defined as genes whose experimental mutation leads to cell death [30]. Furthermore, overlapping *P*. *aeruginosa* GUFs with *P*. *aeruginosa* genes that have homologues only in pathogenic microorganisms (pathogen-associated genes) [31] revealed 28 hits. Four of them (PA0442, PA0977, PA1369, PA2139) are particularly promising targets for a broad range antibiotics, as they were also annotated as generally essential genes (Fig 1B and S1 Table).

Furthermore, we analysed which GUFs might play a role in pathogenic processes by searching for virulence-associated GUFs listed in the virulence factor annotation of the PGD [17]. This list includes genes whose roles in virulence were experimentally proven by studying respective mutant strains or were inferred from sequence similarity with virulence factors from other pathogens. Thirteen identified GUFs were previously linked to virulence-related processes by studying their functions during the infection of model host organisms (e.g., *Caenorhabditis elegans*, *Drosophila melanogaster* or *Rattus norvegicus*), and an additional five were related to common virulence traits (flagella-mediated motility, pyoverdine-mediated iron uptake, type VI secretion of toxins) (S3 Table) [32–36]. Among these virulence-associated GUFs, eleven are conserved in other organisms (S3 Table), suggesting that their analysis might reveal novel insights into general virulence processes in bacteria.

### Assigning putative biochemical functions to *P*. *aeruginosa* proteins of unknown function

To better understand the role of GUFs in *P*. *aeruginosa* infections, it is necessary to know the biochemical functions of proteins encoded by GUFs. We used 3D structure homology modelling as a tool for predicting the biochemical functions of PUFs. This approach relies on the fundamental principle that two homologous proteins with similar sequences have very similar structures. As protein structure is 3–10 times more conserved than the sequence [37], structural modelling can reliably detect homology between proteins with low sequence similarity (<20%) [22]. Knowing that structure determines protein function, one can assume that the functions of the modelled PUF and the template protein with a known structure are likely to be the same. This approach used the advantage of, in most cases, precisely assigned biochemical and even biological functions to proteins with known structures. A further advantage is that generated structural models of target PUFs are useful for the validation of functionally relevant residues, e.g., active site or ligand-binding residues. Additionally, several distinct functional domains in a single target PUF may be identified using different domains with solved structures, and these domains can be combined into a unique multidomain structure. Conclusively, homology modelling of PUF and its comparison with the template may reliably reveal the putative function of PUF.

**Fig 2:**
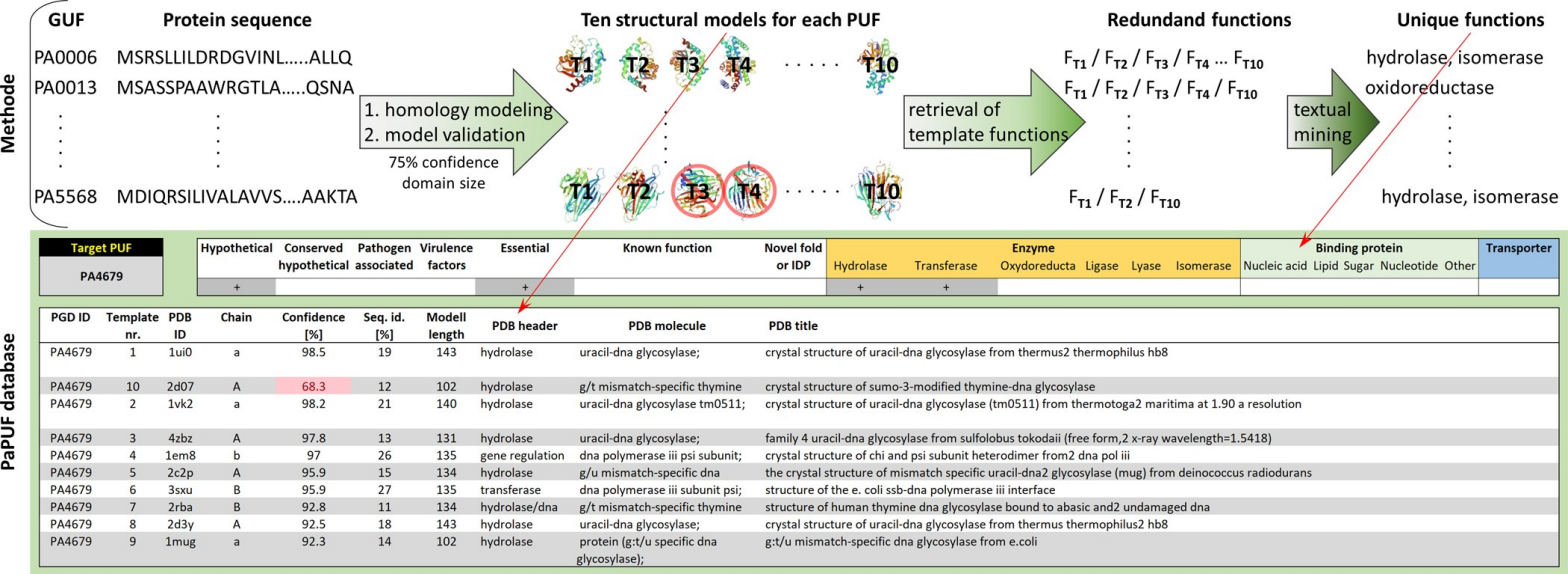
Pipeline for prediction of protein function based on homology modelling with multiple templates. Protein sequences of all 2103 *P*. *aeruginosa* GUFs were retrieved from the UniProt database and submitted to the Phyre2 server to model their structures. Ten structural models for each PUF were validated according to the confidence of homology modelling (>75%) and the size of the modelled domain (>100 residues for transporters and enzymes; >50 residues for binding proteins) to remove unreliable models. Information about the functions of reliable templates was retrieved from their PDB header and PDB molecule fields, and text mining was performed with keywords listed in S6 Table to assign biochemical functions to templates automatically. Redundant functions were removed to compile a list of unique functions of templates that were assigned as putative functions of PUFs. The results are freely available as a downloadable PaPUF database (www.iet.uni-duesseldorf.de/arbeitsgruppen/bacterial-enzymology) in which putative functions of *P*. *aeruginosa* PUFs and ten respective templates can be retrieved by entering a gene name into the “Target PUF” field (upper left corner).

Using protein sequences of PUFs obtained from the UniProt database, we performed homology modelling of 2103 PUFs using the batch homology modelling feature of the Phyre2 server [22]. Retrieving ten models per PUF, we compiled a set of data of approximately 21020 (PA2462, with a size of 573.2 kDa, was too large for homology modelling) homology modelled templates (S4 Table). These results were refined to select reliable models using an automated pipeline (Fig 2). A Phyre confidence score, which indicates a degree of homology between target proteins and the template, was used as the main criterion for the validation of structural prediction. According to this parameter, fairly reliable homology models (>80% conf.) were obtained for most PUFs (75%) (S1A Fig), although their sequence identities were rather low since almost 65% of modelled proteins had sequence identities below 20% compared with the corresponding templates (S2 Fig). Interestingly, 89 PUFs have very low confidences (<20%) (S1 Table), which indicates that these PUFs do not have even remote structural homologues. Hence, these proteins might be intrinsically disordered or have a novel fold and therefore are particularly interesting from the structural biology point of view. Interestingly, we identified 65 PUFs with known structures, as their sequence identities with the template were >96%. Among them were 18 proteins with experimentally demonstrated functions (S5 Table); therefore, they were excluded from our further analysis, and the information is now uploaded to the PGD to improve its annotation.

**Fig 3:**
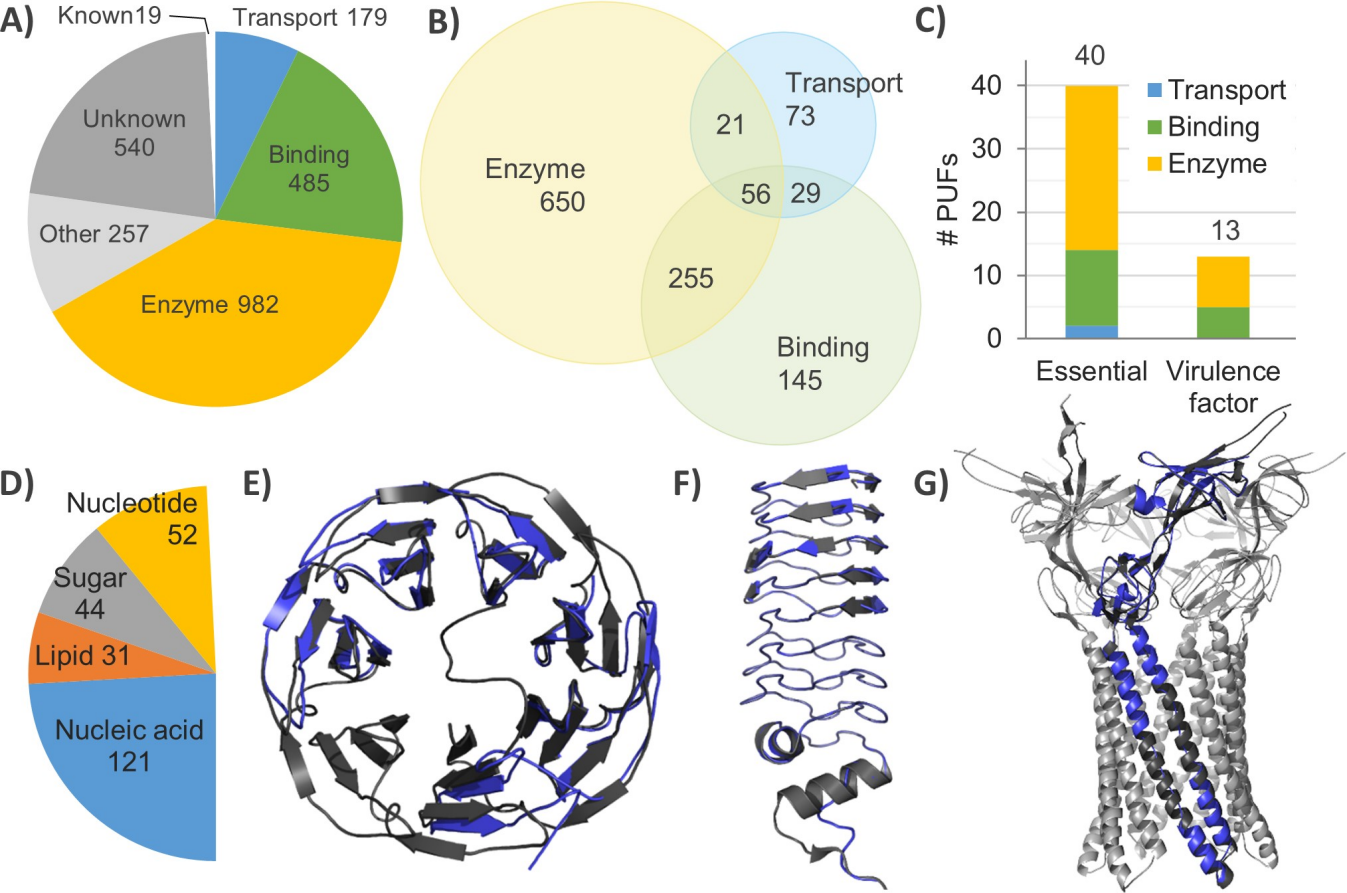
Predicted biochemical functions of *P*. *aeruginosa* proteins of unknown function. **A)** Number of putative enzymes, ligand-binding proteins, transporters, proteins with functions other than the previous three and proteins with known functions. **B)** Venn diagram of PUFs with enzyme, ligand-binding or transport functions. **C)** Distribution of essential and virulence factors among PUFs with the predicted enzyme, ligand-binding or transport functions. Gene identifiers of PUFs with predicted functions (Fig 3A–3C) are provided in S1 Table. **D)** Number of predicted ligand-binding proteins. **E)** Superimposition of a homology model of putative lectin PA5033 with *A*. *aegerita* lectin (PDB ID 4TQJ, chain A, 88.4% conf., 19% seq. id., 1.11 Å rmsd_cα_, 92% coverage, 1.3% of disall. res.). **F)** Superimposition of a homology model of putative DNA gyrase binding protein PA1981 with the *Mycobacterium tuberculosis* MfpA protein (PDB ID 2BM5, chain A, 99% conf., 22% seq. id., 1.45 Å rmsd_cα_, 80% coverage). **G)** Superimposition of a homology model of putative antibiotic efflux pump protein PA3304 with *E*. *coli* MacA (PDB ID 3FPP, chain A, 99% conf., 23% seq. id., 0.56 Å rmsd_cα_, 81% coverage, 1.9% disall. res.). PA3304 is superimposed on one of six MacA protomers, which form a barrel-like functional protein. A homology model is indicated in blue, and the corresponding template grey. The protein structures were visualized using PyMOL software.

To predict the biochemical functions of PUFs with the potential to be novel drug targets, we focused on enzymes, ligand-binding proteins and transporters, which perform indispensable functions for the bacterial cell cycle, infections and antibiotic resistance, e.g., primary metabolism, toxin-mediated host cell lysis, cell surface adhesion and antibiotic expulsion [15, 38, 39]. Consequently, their inhibition might lead to death or reduce the virulence of *P*. *aeruginosa*. To increase the reliability of the functional assignment, we eliminated homology models smaller than 100 residues for enzymes and transporters and smaller than 50 residues for ligand-binding domains. This is in agreement with a thermodynamic folding study that suggests that the optimal size of protein domains is 100 residues [40]. The cut-off for the rejection of binding domains was set to 50 residues, as helix-turn-helix zinc finger DNA-binding domains [41], five-stranded beta-barrel oligonucleotide and oligosaccharide-binding domains [42] and lipid-binding domains [43] of virulence-related proteins are usually not smaller than 50 residues. This comprehensive and accurately refined data set consisting of several reliable protein templates per PUF was a starting point for functional prediction using information about the biochemical functions of the templates. The general text mining algorithm based on the set of keywords (S6 Table), which precisely describe the protein functions studied here, resulted in the prediction of enzyme, binding or transport functions for 59% (1228) of PUFs (Fig 3A). Interestingly, 304 PUFs had two and 59 had three of the analysed functions (Fig 3B).

Unfortunately, the functions of four proteins that belong to *Pseudomonas*-specific and essential protein groups (Fig 1C) were not predicted. Analysis of enzymes, transporters and binding proteins with newly predicted functions revealed that 41 PUFs might be considered potential drug targets, as they were found in essential or virulence-related groups (Fig 3C). Interestingly, comparative sequence analysis revealed that these proteins have 400 homologues (>35% seq. id.) in priority pathogens (S2 Table). Among them are only four proteins with human homologues (S2 Table). Thus, the assignment of putative functions presented here illustrates the global significance for R&D of potential bacteria-specific drug targets.

### Virulence-related functions of putative ligand-binding proteins and transporters

Ligand-binding domains were predicted in 484 proteins, which showed a large overlap (310 proteins) with putative enzymes (Fig 3B). Among them, the proteins predicted to bind nucleic acids were the most abundant (122), followed by nucleotide-binding (52), sugar-binding (44) and lipid-binding (33) proteins (Fig 3D). An interesting example of a virulence-related binding protein is the putative lectin PA5033 (Fig 3E), which was reliably modelled (88% conf.) with the structure of lectin from the mushroom *Agrocybe aegerita* [44]. The results revealed only a small portion (1.3%) of residues in disallowed regions (disall. res.) of the Ramachandran plot as revealed by the stereochemical validation of the energy-minimized PA5033 homology model with PROCHECK (S7 Table) [25]. Hence, most (~80%) high-quality X-ray structures have <5% residues in disallowed regions [45]. This protein, which has experimentally determined extracellular localization, may be involved in bacterial adhesion to host cells or in biofilm formation, as described for the other two (LecA and LecB) extracellular lectins of *P*. *aeruginosa*, which were suggested as targets of biofilm-specific antivirulence drugs [46]. The second interesting candidate is the putative DNA gyrase-binding protein PA1981 (Fig 3F), which was reliably (99% conf.) modelled using the *Mycobacterium tuberculosis* MfpA protein [47]. Proteins from this family bind to DNA gyrase, an antibiotic target with an essential function in the homeostatic control of chromosomal DNA supercoiling, which protects it against the antibiotic fluoroquinolone. Therefore, PA1981 is suggested to be implicated in resistance to fluoroquinolone.

Another example of an antibiotic resistance protein is the putative membrane-bound transporter PA3304, which was reliably modelled with the *E*. *coli* MacA adaptor protein of the efflux pump involved in antibiotic expulsion (Fig 3G and S7 Table) [48]. How many of 105 putative membrane transporters, among 179 predicted transporters in this study (Fig 3A), play a role in antibiotic resistance, tolerance to xenobiotics or the secretion of virulence factors remains to be elucidated.

### Putative enzymatic activities of hypothetical proteins link them to important cellular processes

**Fig 4:**
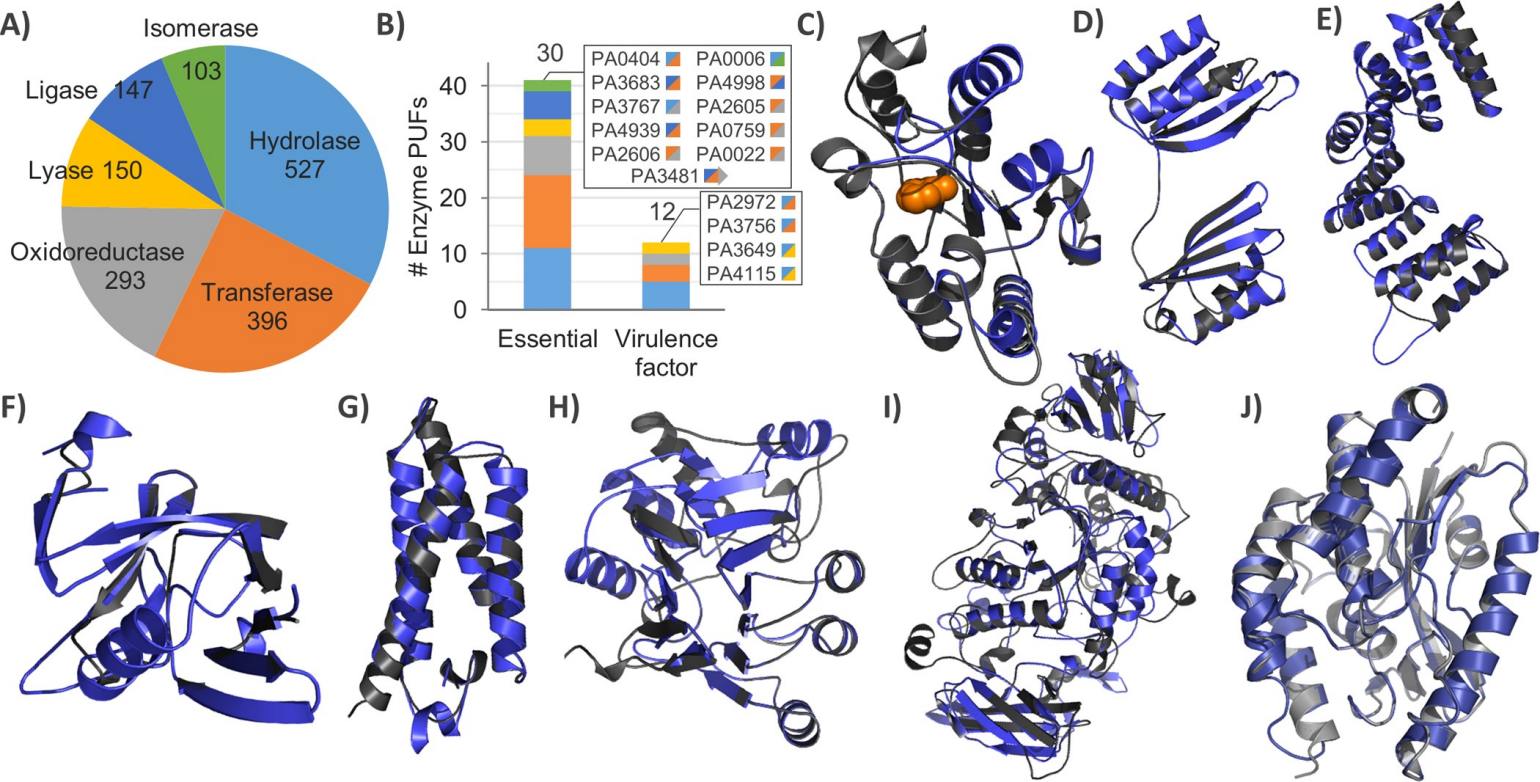
Putative enzymatic activities of *P*. *aeruginosa* proteins of unknown function. **A)** Distribution of putative enzymes among six EC groups. **B)** Distribution of essential genes and virulence factors among GUFs with putative enzyme functions. Gene identifiers of PUFs with predicted functions (Fig 4A and 4B) are provided in S1 Table. **C)** Superimposition of a homology model of putative uracil-DNA glycosylase PA4679 with *T*. *thermophilus* UGD (PDB ID 1UI0, chain A, 98.5% conf., 19% seq. id., rmsd_cα_ = 0.62 Å, 61% coverage, 0.9% disall. res.). The uracil molecule bound to the active site groove of *Tt*UGD is shown as an orange space-filling model. **D)** Superimposition of a homology model of the putative phosphoserine phosphatase PA1009 with *M*. *avium* SerB (PDB ID 3P96, chain A, 99% conf., 12% seq. id., rmsd_cα_ = 0.50 Å, 94% coverage, 1.2% disall. res.). **E)** Superimposition of a homology model of the putative Type IV pili biogenesis protein PA5441 with *N*. *meningitides* PilW (PDB ID 2VQ2, chain A, 93% conf., 17% seq. id., rmsd_cα_ = 0.29 Å, 38% coverage, 0.5% disall. res.). Residues 1–130 of the template are hidden for clarity. **F)** Superimposition of a homology model of the putative L,D-transpeptidase PA3756 with *M*. *tuberculosis* LdtMt2 (PDB ID 3VYN, chain B, 100% conf., 18% seq. id., rmsd_cα_ = 0.65 Å, 83% coverage, 0.8% disall. res.). **G)** Superimposition of a homology model of putative flagellar chaperone PA1095 with *A*. *aeolicus* FliS (PDB ID 1ORJ, chain A, 100% conf., 28% seq. id., rmsd_cα_ = 0.46 Å, 94% coverage, 0% disall. res.). **H)** Superimposition of a homology model of the putative phosphorylcholine esterase PA2984 with *S*. *pneumoniae* Pce (PDB ID 1WRA, chain A, 100% conf., 16% seq. id., rmsd_cα_ = 0.40 Å, 32% coverage, 3% disall. res.). **I)** Superimposition of a homology model of the putative glycoside hydrolase PA2151 with *S*. *pneumoniae* SpuA (PDB ID 2YA0, chain A, 100% conf., 17% seq. id., rmsd_cα_ = 0.54 Å, 94% coverage, 1.9% disall. res.). **J)** Superimposition of a homology model of the putative patatin-like phospholipase PA1640 with *P*. *aeruginosa* outer membrane phospholipase PlpD (PDB ID 5FYA, chain A, 100% conf., 33% seq. id., rmsd_cα_ = 0.36 Å, 72% coverage, 1.4% disall. res.). The homology model is indicated in blue and the corresponding template in grey. The protein structures were visualized using PyMOL (http://www.pymol.org).

Putative enzymes are the largest group (982) of PUFs. Their functional predictions might help to identify potential novel drug targets involved in fundamental metabolic pathways of *P*. *aeruginosa*. The classification of enzymes using text mining revealed hydrolases as the most abundant class, followed by transferases and oxidoreductases (Fig 4A). Thirty-five PUFs with putative enzyme functions are particularly interesting drug targets, as they belong to a group of essential proteins or virulence factors (Fig 4B). Ten of them have two or even three predicted enzyme activities, which indicates profound substrate promiscuity among potential drug targets. However, the multiple predicted functions for these proteins may be caused by using up to ten templates; therefore, the predicted functions should be experimentally validated. An interesting candidate with an essential function is PA4679, a putative uracil-DNA glycosylase (UDG) whose function is predicted based on modelling with *Thermus thermophilus* UGD (Fig 4C). The functional prediction of the UDG activity of PA4579 is strengthened by the finding that the structure of the homology model is similar to that of several other enzymes with UDG activity (root mean square deviation of C_α_ atoms, rmsd_Cα_ < 1.7 Å), and the active site residues of *Tt*UGD that interact with the uracil molecule are conserved among them (Figs 4C and S3 and S8 Table). Its essentiality is consistent with the function of these enzymes in DNA base excision repair, a process essential for maintaining the stability of chromosomal DNA [49].

We predicted the biochemical functions of four PUFs that were previously experimentally determined to have roles in virulence (S3 Table). The putative hydrolase PA1009 was reliably modelled using a template of phosphoserine phosphatase SerB from *Mycobacterium avium*, which is involved in a pyrimidine salvage pathway that is important for genome integrity [50] (Fig 4D and S7 Table). The C-terminal part of the putative cell motility protein PA5441 was reliably modelled with the type IV pili biogenesis protein PilW from *N*. *meningitides*, which plays an important role in the oligomerization and functionality of outer membrane-localized pores of pili [51] (Fig 4E). The putative transferase PA3756 was modelled with 100% confidence using *M*. *tuberculosis* L,D-transpeptidase (LdtMT2), which is a common target of β-lactam antibiotics [52] (Fig 4F and S7 Table). The putative function of PA1095 as a FliS-like chaperone, assigned based on homology with *Aquifex aeolicus* FliS (Fig 4G) [53], is involved in the assembly of the bacterial flagellum. The prediction of virulence functions for PA5441, PA1009, PA3756 and PA1095 is in agreement with the experimental determination of their virulence roles in the genetic screening of mutants in *Rattus norvegicus* virulence assays [54] or motility assays [36].

Furthermore, we predicted the functions of several PUFs based on homology to known virulence factors from other pathogens. Although these proteins were not previously known to be related to virulence in *P*. *aeruginosa*, their putative biochemical functions suggest that they might be novel virulence factors of *P*. *aeruginosa*. Among them are three enzymes, PA2984, PA2151 and PA1640, with putative hydrolytic activities. The C-terminal part (residues 494–736) of PA2984 was reliably modelled (Fig 4H and S7 Table) with phosphorylcholine esterase Pce from *Streptococcus pneumoniae*, whose virulence function is related to adhesion to host cells [55]. The N-terminal domain of PA2984 could not be modelled with any known structure; however, analysis with Phyre2 predicted 12 putative transmembrane helices in this part (S2 Fig), suggesting that this putative hydrolase is a two-domain protein in which the catalytic domain is anchored to the membrane by an N-terminal domain. Interestingly, the *S*. *pneumoniae* template protein also has a second domain that exposes the esterase domain to the cell surface through the binding of surface choline. The structure of the putative hydrolase PA2151 was reliably modelled (Fig 4I and S7 Table) with the glycogen-degrading virulence factor SpuA from *Streptococcus pneumoniae*, which is involved in host-pathogen interactions [56]. The putative extracellular hydrolyse PA1640 was modelled (Fig 4J) with 100% confidence using the structure of type V secreted phospholipase PlpD, which is a putative virulence factor of *P*. *aeruginosa* [57, 58]. PlpD and the *P*. *aeruginosa* type III secretory toxin ExoU [59] belong to the patatin-like family of phospholipases. Patatin domain-containing proteins are very potent toxins due to their strong lytic activity, which they exert on host cells through, among other mechanisms, the hydrolysis of host membrane phospholipids [59].

The functions of the abovementioned enzymes could not be predicted from the sequence alignment, as they share less than 20% sequence identity with the template proteins (except PA1095, which share 28% seg. id.). However, the high confidence of homology modelling for many hypothetical proteins indicates that this comparative approach may be used to predict the biological processes in which they might be involved.

### Conservation of promising drug targets in other pathogens

Protein conservation in multiple pathogens is one of the criteria used to prioritize targets for drug development. Broad-range antibiotics have an advantage over pathogen-specific drugs because of higher profits, easier development due to the uncomplicated recruitment of patients for phase III trials, and no requirement of specific diagnostic tools for pathogen detection [2]. Therefore, we searched orthologues of promising drug targets in clinical isolates of *P*. *aeruginosa*, in other pathogenic *Pseudomonas* species (*P*. *mendocina*, *P*. *otitidis* and *P*. *alcaligenes*), and in pathogens from the WHO priority list (Table 1). The results revealed that each of eleven promising targets with newly predicted ligand binding (Fig 3) or enzyme (Fig 4) functions are conserved (>70% sequence identity, >95% alignment coverage) in most clinical isolates of *P*. *aeruginosa* (Table 1). Furthermore, ten of eleven promising targets are conserved (>70% sequence identity, >95% alignment coverage) in at least two important pathogens from the *Pseudomonas* genus. Finally, most of these eleven proteins might be broad-range targets, as all of them have homologues (nine of eleven proteins were conserved in four or more pathogens) in other WHO priority pathogens (Table 1). Interestingly, seven of these potential drug targets have homologues in both Gram-negative and Gram-positive bacteria (Table 1), indicating that a drug binding these targets might show very broad-spectrum activity. However, this should be experimentally tested, as pathways essential in *P*. *aeruginosa* are not necessarily essential in other pathogens. These proteins whose function was previously unknown do not have human homologues (Table 1), which makes them even more attractive for the development of drugs to combat infections with *Pseudomonas* and other important bacterial pathogens.

**Table 1:** Promising drug targets of *P*. *aeruginosa* among proteins of unknown function.

Conservation	humans	no	no	no	no	no	no	no	no	no	no	no
	clinical *P*. *aeruginosa*^a^	2519	261	2523	1963	2532	2535	2516	1107	1659	2501	653
	pathogenic *Pseudomonas*^b^	*Po*, *Pm*	*Pm*	*Po*, *Pm*, *Pa*	*Po*, *Pm*, *Pa*	*Po*	*Pm*, *Pa*	*Po*, *Pm*	*Po*, *Pm*	*Po*, *Pm*	*Po*, *Pm*	*Pm*, *Pa*
	WHO priority pathogens^c^	*Ab*, *Kp*	*Ab*, ***Sp***, *Ec*, *Sm*, ***Sa***, *Kp*, *St*	*Ab*, *Kp*, *Ec*, *Sf*, *Sm*	*Ab*, *Kp*, *Ec*, *Sf*, *Sm*	*Ab*, *Kp*, *Ec*, *Sm*, ***Sp***, ***Sa***	*Ab*, *Kp*, ***Sp***, *Ec*, ***Ef***	*Ab*, *Kp*, *Ec*, ***Sp***	*Ab*, *Kp*, *Ec*,	*Ab*, *Kp*, ***Sp***, *Ec*	*Ab*, *Kp*, ***Ef***, ***Sp***, *Sm*, ***Sa***	*Ab*, ***Sp***, *Ec*, *Kp*, *Sf*
Phyre 2 confidence [%]		88	99	99	99	93	99	100	94	100	100	100
Putative virulence function		cell adhesion	antibiotic resistance	antibiotic resistance	chromosome stability	cell motility	pyrimidine salvage pathway, genome integrity	peptidoglycan biosynthesis	cell motility	cell adhesion	host-pathogen interactions	secreted toxin
Putative biochemical function		lectin	DNA gyrase-binding protein	efflux pump adapter protein	uracil-DNA glycosylase	Type IV pili protein	phosphoserine phosphatase	L,D-transpeptidases	flagellar chaperon	phosphorylcholine esterase	glycogen-degrading protein	patatin-like phospholipase A
Gene		PA5033	PA1981	PA3304	PA4679	PA5441	PA1009	PA3756	PA1095	PA2984	PA2151	PA1640

^a^Number of orthologues (>70% sequence identity, >95% alignment coverage) identified in strains isolated from humans with the known disease as indicated in the PGD.

^b^Orthologs (>70% sequence identity, >95% alignment coverage) from *P*. *mendocina* (*Pm*), *P*. *otitidis* (*Po*) and *P*. *alcaligenes* (*Pa*), considered pathogenic strains from the *Pseudomonas* genus.

^c^Homologs (>35% sequence identity, >75% alignment coverage) from 13 WHO priority pathogens listed in Fig 1. Gram-positive strains are indicated in bold.

## Discussion

Comparative modelling of the target protein with sequence homologues with known structures is a useful tool for predicting the structures of hypothetical proteins [60–62]. Rarely, homology modelling is used for proteome-wide structural and functional annotation of hypothetical proteins [63, 64]. Here, we have demonstrated the applicability of multitemplate homology modelling for the global assignment of biochemical functions to hundreds of hypothetical proteins of the human pathogen *P*. *aeruginosa* to aid the identification of potential antibiotic targets. For that purpose, we used the Phyre2 server, which is among the most commonly used tools for the prediction of protein structure [22]. Phyre2 applies an advanced method for the detection of remote sequence homologues with high specificity and sensitivity to build an accurate model even for proteins with less than 20% sequence identity [22]. In contrast to functional prediction based on pairwise sequence alignment, which appears to be reliable only when sequence similarity is very high because homologous genes can diverge to have different functions [65, 66], structure-based functional predictions take advantage of the fact that the protein structure remains conserved long after sequences are substantially changed [67]. Conclusively, Phyre2 functional annotation shows an advantage over sequence-alone homology searches.

Our set of >21000 homology models for >2100 target *P*. *aeruginosa* PUFs generated using homologues with known structures and in most cases experimentally determined biochemical functions was refined to identify true homologues. The biochemical functions of true template homologues were automatically assigned as putative functions to hundreds of target proteins. The results of this functional prediction are available as the PaPUF database (www.iet.uni-duesseldorf.de/arbeitsgruppen/bacterial-enzymology). It is likely that for some PaPUFs, homology-modelling-based functional assignment is misleading because it is known that during evolution from one protein, several homologues may diverge to perform different functions [68]. Therefore, to reinforce the prediction of biochemical functions, we recommend verifying the biochemical functions by superimposing the homology model with the template homologues and inspecting active sites or ligand binding sites. However, this process is tedious and time consuming and therefore is not applicable for high-throughput analysis. Our approach revealed 360 PUFs with two or three putative biochemical functions, which were associated with different template structures. This is not surprising if we consider that many enzymes catalyse more than one reaction [69–71], that many enzymes and transporter domains have annotated ligand-binding domains [72–75], and that several distinct domains are often arranged into one functional protein [76, 77].

Although biochemical function is an important prerequisite for drug R&D, it is not sufficient alone, without biological function, for identifying potential drug targets [78, 79]. Therefore, the putative biological functions of the template homologues were used to predict the roles of PUFs in virulence and antibiotic resistance. During the selection of putative drug targets, we considered information obtained from large-scale screens [30, 32–36] about the relationship of target *P*. *aeruginosa* PUFs with virulence or essential processes, the conservation of PUFs in other high-priority relevant pathogens and the absence of PUF homologues in humans. This led to the classification of 107 PUFs (63 essential, 21 virulence factor, 28 pathogen-specific) into the highest priority group for the development of novel drugs against *P*. *aeruginosa*. Notably, 103 of these PUFs do not have a human homologue, which is a good starting point for the development of selective drugs. Forty-one of these top priority drug targets were assigned putative biochemical functions with high homology modelling confidence, query protein coverage and good stereochemical parameters. These functions allowed us, based on the biological processes ascribed to homology templates, to link them with maintaining genome integrity (putative phosphoserine phosphatase PA1009 and putative uracil-DNA glycosylase PA4679), peptidoglycan biosynthesis (putative L,D-transpeptidase PA3756), host-pathogen interactions (putative extracellular lectin PA5033, putative phosphorylcholine esterase PA2984, putative flagellar chaperone PA1095, putative Type IV pili biogenesis protein PA5441 and putative glycogen-degrading protein PA2151), extracellular toxicity (patatin-like phospholipase PA1640) and antibiotic resistance (putative DNA gyrase binding protein PA1981 and putative efflux pump adapter protein PA3304) (Table 1). Interestingly, several of these drug targets were previously identified as virulence-related genes using a *Rattus norvegicus* virulence assay [54] or motility assay [36], although their functions were unknown.

All these processes are common drug targets; therefore, we believe that these proteins are promising targets for further experimental characterization and the design of inhibitors, which might represent novel antibiotics to treat not only *P*. *aeruginosa* but also other high-priority pathogens in which these proteins are largely conserved.

## Supporting information

S1 Fig Confidence score (A) and sequence identity (B) distributions for homology modelling of all 2102 P. aeruginosa PUFs obtained with the use of Phyre2 server. The only highest confidence score of each PUF was considered in the analysis.
(TIF)

S2 Fig Topology of putative transmembrane helices predicted in PA2984 by Phyre2 server. (TIF)

S3 Fig Conservation of functional residues among PaPUFs and their structural homologs obtained by DALI database [1]. Literature reported active site and ligand binding residues are highlighted gray. Superimposition parameters are provided in S8 Table.
(TIF)

S1 Table Classification of *P*. *aeruginosa* GUFs. (XLSX)

S2 Table Priority pathogens analyzed in this study. (DOCX)

S3 Table *P*. *aeruginosa* GUFs with experimentally demonstrated virulence function. (DOCX)

S4 Table Homology modelling of *P*. *aeruginosa* PUFs using Phyre2 server. (XLSX)

S5 Table PUFs with more than 96% of sequence identity to templates with known function. (DOCX)

S6 Table Keywords used for textual mining of enzyme, ligand binding and transporter functions of templates used for modelling of *P*. *aeruginosa* PUFs. (DOCX)

S7 Table Stereochemical validation of PaPUF homology models with PROCHECK [11]. (DOCX)

S8 Table Structural alignments of PUF homology model with its corresponding template and additional proteins belonging to the same functional class, according to DALI database. (DOCX)

S1 File References specific to supporting information. (DOCX)
