# Supplementary figures and images for: Predicting drug targets by homology modelling of Pseudomonas aeruginosa proteins of unknown function
Source: PLoS One. 2021 Oct 14;16(10):e0258385. doi: 10.1371/journal.pone.0258385 (PMC8516228; doi:10.1371/journal.pone.0258385)

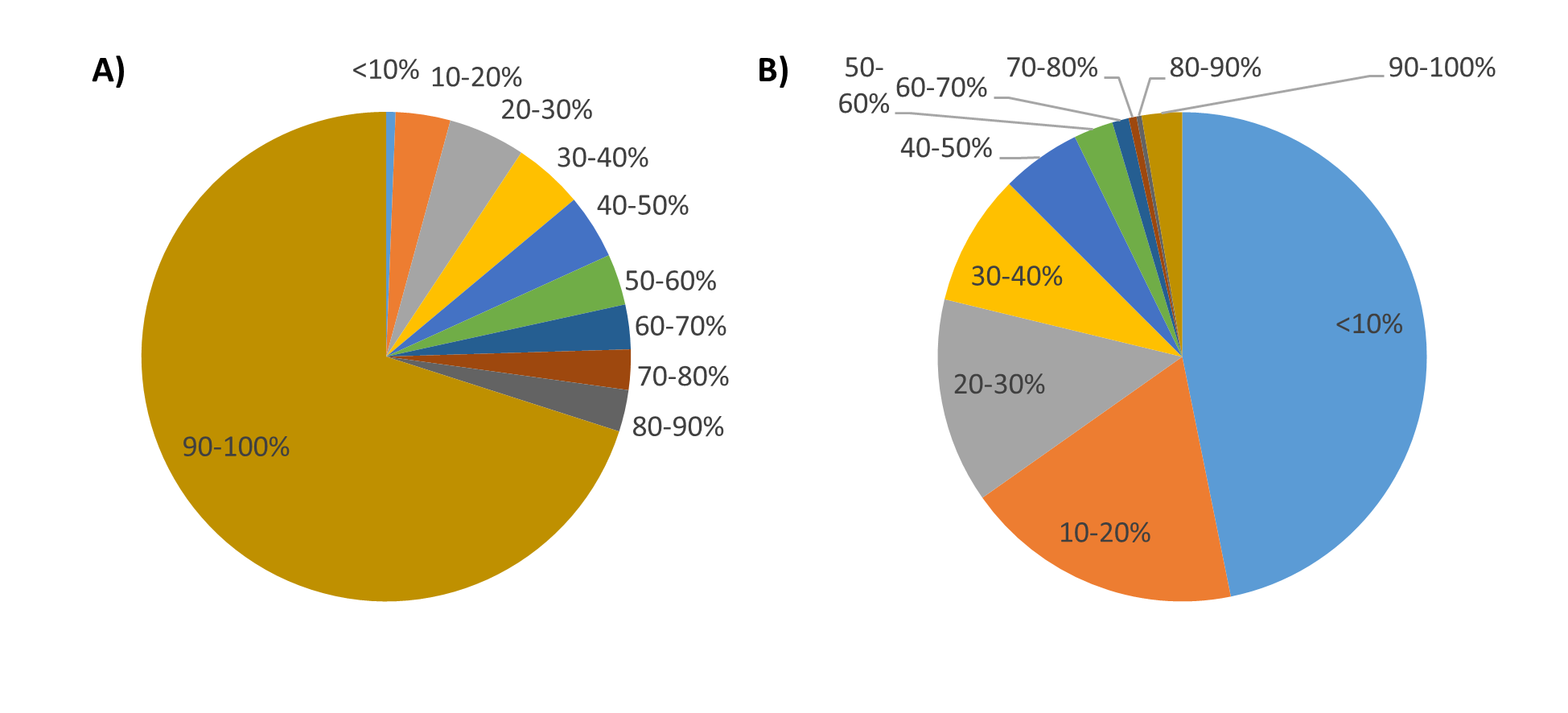

Supplement: S1 Fig — Confidence score (A) and sequence identity (B) distributions for homology modelling of all 2102 P. aeruginosa PUFs obtained with the use of Phyre2 server. The only highest confidence score of each PUF was considered in the analysis. (TIF) [file pone.0258385.s001.tif]

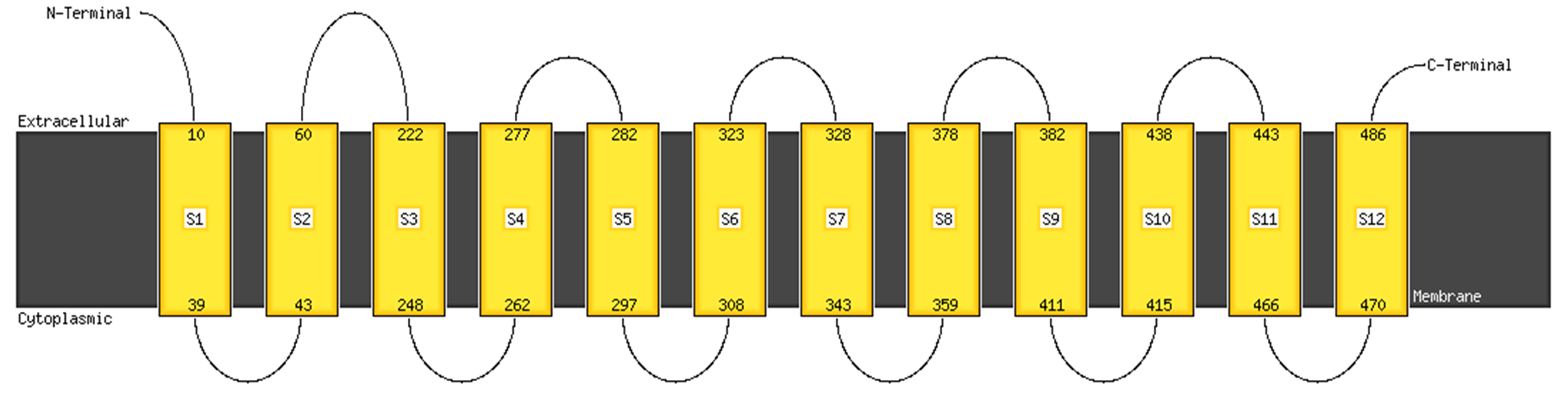

Supplement: S2 Fig — (TIF) [file pone.0258385.s002.tif]

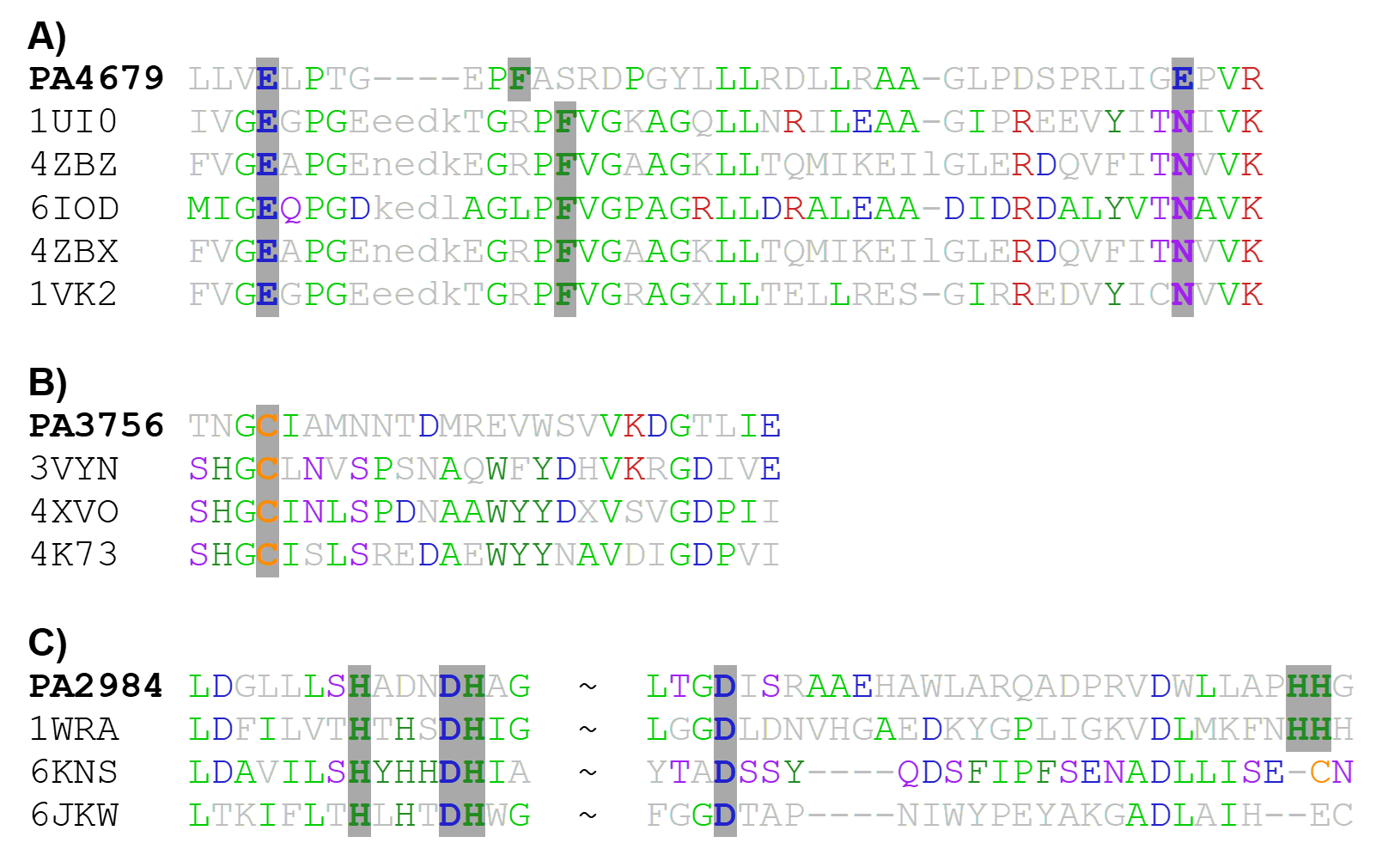

Supplement: S3 Fig — Literature reported active site and ligand binding residues are highlighted gray. Superimposition parameters are provided in S8 Table. (TIF) [file pone.0258385.s003.tif]
