# Supplementary material for: Predicting drug targets by homology modelling of Pseudomonas aeruginosa proteins of unknown function
Source: PLoS One. 2021 Oct 14;16(10):e0258385. doi: 10.1371/journal.pone.0258385 (PMC8516228; doi:10.1371/journal.pone.0258385)
Supplement: S2 Table — (DOCX) [file pone.0258385.s005.docx]

**S2 Table:** Priority pathogens analyzed in this study.

| Organism | Taxonomic identifier | Nr. of homologs of 41 PUFs* |
| --- | --- | --- |
| *Acinetobacter baumanii* | 470 | 41 |
| *Klebsiella pneumoniae* | 573 | 40 |
| *Escherichia coli* | 562 | 41 |
| *Serratia marcescens* | 615 | 33 |
| *Campylobacter sp.* AG18-0001 | 197 | 32 |
| *Salmonella typhi* | 90370 | 30 |
| *Staphylococcus aureus* | 1280 | 25 |
| *Neisseria gonorrhoeae* | 485 | 25 |
| *Enterococcus faecium* | 1352 | 25 |
| *Helicobacter pylori* | 210 | 16 |
| *Streptococcus pneumoniae* | 1313 | 38 |
| *Shigella flexneri* | 623 | 31 |
| *Haemophilus influenzae* | 727 | 23 |

*Virulence-related and essential PUFs with predicted biochemical functions were used as a query for BLAST search on NCBI.
