# Supplementary material for: Predicting drug targets by homology modelling of Pseudomonas aeruginosa proteins of unknown function
Source: PLoS One. 2021 Oct 14;16(10):e0258385. doi: 10.1371/journal.pone.0258385 (PMC8516228; doi:10.1371/journal.pone.0258385)
Supplement: S3 Table — (DOCX) [file pone.0258385.s006.docx]

**S3 Table:** *P. aeruginosa* GUFs with experimentally demonstrated virulence function.

| **PGD ID** | **GUF category** | **Virulence process** | **Reference** |
| --- | --- | --- | --- |
| PA2146 | conserved hypothetical | *Hordeum vulgare* | [6] |
| PA2360 | hypothetical | *Caenorhabditis elegans* | [7] |
| PA2372 | hypothetical | *Caenorhabditis elegans* | [7] |
| PA2827 | conserved hypothetical | *Drosophila melanogaster* | [8] |
| PA1009 | hypothetical | *Rattus norvegicus* | [9] |
| PA2972 | conserved hypothetical | *Rattus norvegicus* | [9] |
| PA3756 | hypothetical | *Rattus norvegicus* | [9] |
| PA3826 | hypothetical | *Rattus norvegicus* | [9] |
| PA4115 | conserved hypothetical | *Rattus norvegicus* | [9] |
| PA4564 | conserved hypothetical | *Rattus norvegicus* | [9] |
| PA4692 | conserved hypothetical | *Rattus norvegicus* | [9] |
| PA5441 | hypothetical | *Rattus norvegicus* | [9] |
| PA2146 | conserved hypothetical | *Hordeum vulgare* | [6] |
| PA2462 | hypothetical | Hemolysis | [6] |
| PA1093 | hypothetical | Flagellum function | [10] |
| PA1095 | hypothetical | Flagelum function | [10] |
| PA1096 | hypothetical | Flagelum function | [10] |
| PA1442 | conserved hypothetical | Flagelum function | [10] |
