# Supplementary material for: Predicting drug targets by homology modelling of Pseudomonas aeruginosa proteins of unknown function
Source: PLoS One. 2021 Oct 14;16(10):e0258385. doi: 10.1371/journal.pone.0258385 (PMC8516228; doi:10.1371/journal.pone.0258385)
Supplement: S5 Table — (DOCX) [file pone.0258385.s008.docx]

**S5 Table:** PUFs with more than 96% of sequence identity to templates with known function.

| **PA Nr** | **Confidence (%)** | **Sequence identity (%)** | **Query coverage*** | **PDB ID of template** | **Function** | **PubMed ID** |
| --- | --- | --- | --- | --- | --- | --- |
| PA0666 | 100,0 | 100 | 360 | 3qbw | 1,6-Anhydro-N-acetylmuramic acid kinase | 21288904 |
| PA3800 | 100,0 | 100 | 352 | 4hdj | BamB, component of β-Barrel Assembly Machine (BAM) | 23189157 |
| PA3086 | 99,5 | 100 | 69 | 2gqc | protease | 17059825 |
| PA4534 | 100,0 | 100 | 136 | 4ubr | N-acetyltransferase | to be published |
| PA4279 | 100,0 | 98 | 245 | 2f9t | transferase | 16905099 |
| PA3263 | 100,0 | 100 | 305 | 2owy | recombination-associated protein RdgC | 17426134 |
| PA3302 | 100,0 | 100 | 154 | 5cpg | lyase | 26386053 |
| PA4991 | 100,0 | 98 | 390 | 5ez7 | FAD dependent oxidoreductase | 26841760 |
| PA3764 | 100,0 | 97 | 425 | 4oz9 | lyase | 27618662 |
| PA0616 | 100,0 | 100 | 174 | 4s37 | R2 pyocin membrane-piercing spike | to be published |
| PA0115 | 100,0 | 98 | 148 | 1xeb | Acyl-CoA N-acyltransferase | to be published |
| PA5396 | 100,0 | 97 | 324 | 2i5g | amidohydrolase | to be published |
| PA5201 | 100,0 | 96 | 323 | 3bzk | transcription Tex protein | 18321528 |
| PA1221 | 100,0 | 99 | 586 | 4dg9 | ligase | 22452656 |
| PA5185 | 100,0 | 100 | 138 | 2o5u | thioesterase | 19898606 |
| PA1865 | 100,0 | 100 | 537 | 4r8a | hydrolase | 25319828 |
| PA4992 | 100,0 | 100 | 267 | 4exa | Aldo_ket_red domain-containing protein | 23295481 |
| PA4872 | 100,0 | 100 | 283 | 3b8i | oxaloacetate decarboxylase | 18081320 |

*Number of residues.
