# Supplementary material for: Predicting drug targets by homology modelling of Pseudomonas aeruginosa proteins of unknown function
Source: PLoS One. 2021 Oct 14;16(10):e0258385. doi: 10.1371/journal.pone.0258385 (PMC8516228; doi:10.1371/journal.pone.0258385)
Supplement: S6 Table — (DOCX) [file pone.0258385.s009.docx]

**S6 Table:** Keywords used for textual mining of enzyme, ligand binding and transporter functions of templates used for modelling of *P. aeruginosa* PUFs.

| **Hydrolase** | **Transferase** | **Oxidoreductase** | **Ligase** | **Lyase** | **Isomerase** |
| --- | --- | --- | --- | --- | --- |
| *hydrolase* | *transferase* | *oxidoreductase* | *ligase* | *lyase* | *isomerase* |
| *cellulase* | *kinase* | *hydroxylase* | *synthetase* | *aldolase* | *epimerase* |
| *glucosidase* | *phosphorylase* | *reductase* | *synthase* | *fumarse* | *mutase* |
| *gtpase* | *transaldolase* | *oxidase* |  | *cyclase* | *racemase* |
| *atpase* | *transglutaminase* | *oxido-reductase* |  | *dehydrochlorinase* |  |
| *peptide release* | *polymerase* | *flavoenzyme* |  |  |  |
| *peptidase* | *thiolase* | *hydrogenase* |  |  |  |
| *nuclease* |  | *oxygenase* |  |  |  |
| *dnase* |  | *cytochrom* |  |  |  |
| *rnase* |  |  |  |  |  |
| *phosphatase* |  |  |  |  |  |
| *amylase* |  |  |  |  |  |
| *amidase* |  |  |  |  |  |
| *esterase* |  |  |  |  |  |
| *protease* |  |  |  |  |  |
| *proteinase* |  |  |  |  |  |
| *helicase* |  |  |  |  |  |
| *leishmanolysin* |  |  |  |  |  |
| *hydrolytic enzyme* |  |  |  |  |  |
| *lipase* |  |  |  |  |  |
| *phospholipase* |  |  |  |  |  |
| **Binding** | | | | **Transporter** | |
| **Nucleic acids** | **Lipid** | **Sugar** | **Nucleotid** |  | |
| *dna* | *lipid* | *cellulose* | *atp* | *porin* | *export* |
| *rna* | *fatty acid* | *carbohydrat* | *amp* | *channel* | *import* |
| *nucleic* |  | *peptidoglycan* | *nucleot* | *efflux* | *uptake* |
|  |  | *lectin* | *fmn* | *pump* | *permease* |
|  |  | *sugar* |  | *transporter* | *anti/sim/porter* |
|  |  | *maltose* |  |  |  |

* indicate any character
