# Supplementary material for: Predicting drug targets by homology modelling of Pseudomonas aeruginosa proteins of unknown function
Source: PLoS One. 2021 Oct 14;16(10):e0258385. doi: 10.1371/journal.pone.0258385 (PMC8516228; doi:10.1371/journal.pone.0258385)
Supplement: S7 Table — (DOCX) [file pone.0258385.s010.docx]

**S7 Table:** Stereochemical validation of PaPUF homology models with PROCHECK. [11]

| **PaPUF** | **Core (%)** | **Allowed (%)** | **General (%)** | **Disallowed (%)** |
| --- | --- | --- | --- | --- |
| PA2151 | 81.0 | 14.7 | 2.3 | 1.9 |
| PA2984 | 80.5 | 14.5 | 2.0 | 3.0 |
| PA5033 | 73.6 | 22.0 | 3.1 | 1.3 |
| PA1095 | 89.4 | 9.6 | 1.0 | 0.0 |
| PA3756 | 79.7 | 16.1 | 3.4 | 0.8 |
| PA1009 | 85.2 | 13.6 | 0.0 | 1.2 |
| PA4679 | 83.8 | 12.0 | 3.4 | 0.9 |
| PA3304 | 78.3 | 16.5 | 3.3 | 1.9 |
| PA1981 | 72.7 | 26.0 | 0.6 | 0.6 |
| PA5441 | 91.4 | 8.1 | 0.0 | 0.5 |
| PA1640 | 86.6 | 11.5 | 0.5 | 1.4 |
