# Supplementary material for: Predicting drug targets by homology modelling of Pseudomonas aeruginosa proteins of unknown function
Source: PLoS One. 2021 Oct 14;16(10):e0258385. doi: 10.1371/journal.pone.0258385 (PMC8516228; doi:10.1371/journal.pone.0258385)
Supplement: S8 Table — (DOCX) [file pone.0258385.s011.docx]

**S8 Table.** Structural alignments of PUF homology model with its corresponding template and additional proteins belonging to the same functional class, according to DALI database.

| **PaPUF** | **Phyre2 template***  **PDB ID / rmsd [Å]** | **Homolog 1**  **PDB ID / rmsd [Å]** | **Homolog 2**  **PDB ID / rmsd [Å]** | **Homolog 3**  **PDB ID / rmsd [Å]** | **Homolog 4**  **PDB ID / rmsd [Å]** |
| --- | --- | --- | --- | --- | --- |
| PA2984 | 1WRA / 1.2 | 6KNS / 3.2 | 6JKW / 3.3 | - | - |
| PA3756 | 3VYN / 0.6 | 4XVO / 1.7 | 4K73 / 1.3 | - | - |
| PA4679 | 1UI0 / 0.8 | 4ZBZ / 1.4 | 6IOD / 1.7 | 4ZBX / 1.4 | 1VK2 / 1.4 |
