# Supplementary material for: Predicting drug targets by homology modelling of Pseudomonas aeruginosa proteins of unknown function
Source: PLoS One. 2021 Oct 14;16(10):e0258385. doi: 10.1371/journal.pone.0258385 (PMC8516228; doi:10.1371/journal.pone.0258385)
Supplement: S1 File — (DOCX) [file pone.0258385.s012.docx]

**References to supporting information of the article:**

**Predicting drug targets by homology modelling of *Pseudomonas aeruginosa* proteins of unknown function**

Nikolina Babic^1^ and Filip Kovacic^1,^*

1. Institute of Molecular Enzyme Technology, Heinrich-Heine Universität Düsseldorf, Forschungszentrum Jülich, D-52426 Jülich, Germany

*corresponding author: Filip Kovacic, Institute of Molecular Enzyme Technology, Heinrich-Heine Universität Düsseldorf, Forschungszentrum Jülich, D-52426 Jülich, Germany, [f.kovacic@fz-juelich.de](mailto:f.kovacic@fz-juelich.de)

**References to supporting information:**

[1] L. Holm, P. Rosenstrom, Dali server: conservation mapping in 3D, Nucleic Acids Res., 38 (2010) W545-549.

[2] S.J.H. Sui, A. Fedynak, W.W. Hsiao, M.G. Langille, F.S. Brinkman, The association of virulence factors with genomic islands, PloS one, 4 (2009) e8094.

[3] B. Liu, D. Zheng, Q. Jin, L. Chen, J. Yang, VFDB 2019: a comparative pathogenomic platform with an interactive web interface, Nucleic acids research, 47 (2019) D687-D692.

[4] S. Sayers, L. Li, E. Ong, S. Deng, G. Fu, Y. Lin, B. Yang, S. Zhang, Z. Fa, B. Zhao, Victors: a web-based knowledge base of virulence factors in human and animal pathogens, Nucleic acids research, 47 (2019) D693-D700.

[5] S.A. Lee, L.A. Gallagher, M. Thongdee, B.J. Staudinger, S. Lippman, P.K. Singh, C. Manoil, General and condition-specific essential functions of Pseudomonas aeruginosa, Proceedings of the National Academy of Sciences, 112 (2015) 5189-5194.

[6] C. Attila, A. Ueda, S.L. Cirillo, J.D. Cirillo, W. Chen, T.K. Wood, Pseudomonas aeruginosa PAO1 virulence factors and poplar tree response in the rhizosphere, Microbial biotechnology, 1 (2008) 17-29.

[7] R.L. Feinbaum, J.M. Urbach, N.T. Liberati, S. Djonovic, A. Adonizio, A.-R. Carvunis, F.M. Ausubel, Genome-wide identification of Pseudomonas aeruginosa virulence-related genes using a Caenorhabditis elegans infection model, PLoS pathogens, 8 (2012) e1002813.

[8] A. Romsang, S. Atichartpongkul, W. Trinachartvanit, P. Vattanaviboon, S. Mongkolsuk, Gene expression and physiological role of Pseudomonas aeruginosa methionine sulfoxide reductases during oxidative stress, Journal of bacteriology, 195 (2013) 3299-3308.

[9] E. Potvin, D.E. Lehoux, I. Kukavica‐Ibrulj, K.L. Richard, F. Sanschagrin, G.W. Lau, R.C. Levesque, In vivo functional genomics of Pseudomonas aeruginosa for high‐throughput screening of new virulence factors and antibacterial targets, Environmental microbiology, 5 (2003) 1294-1308.

[10] N. Dasgupta, M.C. Wolfgang, A.L. Goodman, S.K. Arora, J. Jyot, S. Lory, R. Ramphal, A four‐tiered transcriptional regulatory circuit controls flagellar biogenesis in Pseudomonas aeruginosa, Mol Microbiol, 50 (2003) 809-824.

[11] R.A. Laskowski, M.W. MacArthur, D.S. Moss, J.M. Thornton, PROCHECK: a program to check the stereochemical quality of protein structures, Journal of applied crystallography, 26 (1993) 283-291.
